# Dermal Fibroblast Senescence: The Central Hub of Skin Aging—From Intrinsic Dysfunction to Microenvironmental Remodeling

**DOI:** 10.3390/ijms27041653

**Published:** 2026-02-08

**Authors:** Jinyu Zheng, Sensen Wang, Jiaming Sun, Jingwei Lv

**Affiliations:** School of Pharmacy, Changchun University of Chinese Medicine, Changchun 130117, China18834062064@163.com (S.W.)

**Keywords:** skin aging, dermal fibroblasts, extracellular matrix, intercellular communication, senescence-associated secretory phenotype

## Abstract

Skin aging commonly manifests as deepening wrinkles, loss of elasticity, and weakened barrier function, resulting from the long-term accumulation of multiple biological processes. Dermal fibroblasts, as the primary source of extracellular matrix, not only provide structural support but also play an active role in aging. On one hand, they undergo intrinsic aging due to telomere shortening, mitochondrial decline, and dysregulation of signaling pathways (e.g., TGF-β, mTOR). On the other hand, they release inflammatory cytokines and proteases via the senescence-associated secretory pattern (SASP), disrupting keratinocyte function, melanin distribution, immune surveillance, and even microvascular and adipose tissue functions. This destabilizes the matrix equilibrium and exacerbates inflammation, creating a vicious cycle. While strategies like dasatinib/quercetin, rapamycin, or retinol show promise, they remain constrained by transdermal efficiency and targeting limitations. This review aims to elucidate these mechanisms and interactions, providing insights for developing more effective anti-aging interventions.

## 1. Introduction

It is projected by the United Nations World Population Prospects that by 2024, close to 10% of the global population, or about 830 million people, will be aged 65 or above. The proportion is expected to double by 2050, making up nearly 20% of the global population, which is about 1.7 billion people. As the global population ages more rapidly, it is anticipated that by 2050, individuals aged 65 and above will make up roughly a quarter of the population in numerous countries [1]. One of the most prominent features of systemic aging is skin aging, manifested as age-related degenerative changes in skin structure and function. Clinically, these changes appear as decreased elasticity, increased wrinkling, and heightened pigmentation [2]. These alterations not only affect appearance but may also disturb self-perception, social behavior, and mental health, thereby increasing the risk of emotional disorders, including anxiety and depression [3]. Furthermore, continuous skin aging is connected to the onset of chronic inflammatory dermatoses, including atopic dermatitis, chronic spontaneous urticaria, and bullous pemphigoid, along with systemic inflammatory aging [4]. The local microenvironment of aging skin exhibits pathological characteristics highly similar to those of chronic, hard-to-heal wounds, including persistent low-grade inflammation, excessive accumulation of reactive oxygen species (ROS), and tissue hypoxia. Recent studies on diabetic wounds indicate that this microenvironment significantly suppresses tissue repair capacity and promotes extracellular matrix degradation by forming a vicious cycle of “ROS-inflammation-hypoxia” [5]. Furthermore, microbial colonization (such as methicillin-resistant Staphylococcus aureus) and its biofilm formation not only constitute physical and biochemical barriers but also sustain activation of local immune responses, further impeding the regenerative process [6]. These observations suggest that skin aging is not solely attributable to intrinsic cellular senescence but is also driven by persistent alterations in the microenvironment. Therefore, gaining a deep understanding of the molecular mechanisms underlying skin aging has become a central focus in anti-aging research, and the role of dermal fibroblasts has also garnered increasing attention.

As the primary cells responsible for collagen synthesis and remodeling, the functional decline of these cells has long been recognized as a key factor in collagen loss within aging skin [7,8]. However, research over the past decade has revealed that fibroblasts are far from passive victims in the aging process: under ultraviolet irradiation or age-related stress, they can enter a stable state of cell cycle arrest (i.e., cellular senescence), a process jointly mediated by telomere attrition, mitochondrial dysfunction, cumulative DNA damage, and epigenetic reprogramming. More significantly, senescent fibroblasts secrete large amounts of inflammatory cytokines (such as IL-6 and IL-8), matrix metalloproteinases (MMPs), and other signaling molecules, forming the characteristic senescence-associated secretory phenotype (SASP) [9,10]. Although SASP may participate in repair during acute injury, its prolonged persistence suppresses keratinocyte proliferation through paracrine effects, disrupts melanin distribution, weakens local immune surveillance, and exacerbates ECM degradation, thereby establishing a self-sustaining pro-inflammatory microenvironment within the skin [9,11].

Existing research predominantly focuses on clinical phenotypes of skin aging, individual signaling pathways (such as TGF-β or NF-κB), or enumerating components of the senescence-associated secretory phenotype (SASP). There is limited systematic integration of intrinsic aging mechanisms in fibroblasts—including mitochondrial dysfunction and epigenetic reprogramming—cross-cell effects (involving epidermal barrier integrity, immune polarization, microvascular stability, and subcutaneous fat homeostasis), and the efficacy and limitations of intervention strategies. This paper traces the logical connections from molecular drivers to tissue interactions and intervention strategies, focusing on fibroblasts’ dual role in skin aging—both responding to and sustaining cell-autonomous aging programs while actively shaping pro-inflammatory microenvironments. It provides a mechanistically coherent analytical framework for understanding the systemic characteristics of skin aging.

## 2. Manifestations and Types of Skin Aging

The skin is the largest organ in the human body, composed of three layers: the epidermis, dermis, and subcutaneous tissue. Together, they maintain critical functions such as barrier protection, support, sensation, and immunity [12,13]. With advancing age, these three layers undergo progressive disruption at the cellular and molecular levels—including the decline of epidermal stem cell function, degradation of the dermal extracellular matrix (ECM), and atrophy of subcutaneous fat—ultimately manifesting as macroscopic signs of aging such as wrinkles, sagging, pigmentation abnormalities, dryness, and diminished repair capacity [14,15]. It is noteworthy that these phenotypes are not the result of a single process, but rather the manifestation of the interplay between endogenous (chronological) and exogenous (environmental) aging mechanisms. One of the core driving factors is the entry of key cells—specifically fibroblasts—into a senescent state. In this systematic exposition of clinical manifestations of skin aging, we first establish the biological definition of “cellular senescence” and thoroughly analyze its specific expressions within the epidermis, dermis, and subcutaneous tissue. Finally, we delineate distinctions between intrinsic and extrinsic aging. By integrating evidence from histological, molecular, and clinical perspectives, this work lays the groundwork for subsequent in-depth exploration of the mechanisms underlying fibroblast senescence.

### 2.1. Manifestations of Skin Aging

#### 2.1.1. Definition of Aging

In skin aging research, “senescence” specifically refers to cellular senescence. This is a stable and irreversible state of cell cycle arrest induced by multiple stressors, including DNA damage, oxidative stress, nutrient deprivation, hypoxia, mitochondrial dysfunction, and oncogene activation [16]. Despite losing their proliferative capacity, senescent cells maintain active metabolic functions and continue to influence the surrounding microenvironment through paracrine signaling. Notably, these cells are not quiescent but significantly alter local tissue homeostasis by secreting active molecules such as inflammatory mediators, proteases, and growth factors. In skin, chronic UV exposure and persistent oxidative stress represent the primary exogenous drivers inducing dermal fibroblasts into a senescent state.

#### 2.1.2. Manifestations of Aging in the Epidermis and Subcutaneous Tissue

In the epidermis and dermis, melanocytes and fibroblasts are widely regarded as key cell types involved in human skin aging [17]. Keratinocytes mainly make up the epidermis, undergoing final differentiation to create a layered, stratified structure. The outermost layer forms a physical barrier made of keratin and intercellular lipids that protects against chemical, physical, and microbial insults from the environment. Research has shown that epidermal stem cells have specific regenerative properties that play a role in the aging of the skin. Research has demonstrated that with age, the stem cells in the basal layer of the epidermis experience a decline in their capacity to proliferate and differentiate. This decline has been associated with epidermal thinning, slower renewal rates, and impaired barrier function [18,19].

Compared with epidermal stem cells, dermal fibroblasts have been reported to show a stronger response to aging. Consequently, age-related changes in the dermis are considered to play an important role in skin aging. Among the hallmark manifestations of skin aging, wrinkles and reduced elasticity are largely attributed to progressive dermal atrophy [12]. The dermis includes a variety of stromal cells along with the extracellular matrix [20]. The ECM consists largely of structural proteins (such as collagen and elastin), glycoproteins (such as fibronectin and laminin), proteoglycans, and glycosaminoglycans. The ECM forms a three-dimensional network through interactions among its molecules, including collagen, elastin, and fibronectin, which provides the skin with structural support, elasticity, and flexibility [21,22,23]. Young skin features an abundance of densely packed and well-structured collagen fibers, while aged skin shows more fragmentation and disorganization, along with a reduced amount of collagen [24,25]. Other ECM components also undergo age-related alterations, which contribute to dermal atrophy and decreased mechanical strength. Dysfunction of subcutaneous adipose tissue has been proposed as an early indicator of accelerated skin aging. In aged skin, reductions in adipocyte number are associated with a lower overall proportion of subcutaneous tissue lipids. These changes may compromise structural support of the skin [26,27].

Skin aging involves coordinated alterations in the epidermis, dermis, and subcutaneous tissue. Diminished function of epidermal stem cells weakens barrier maintenance and regenerative capacity, while dermal fibroblast senescence leads to persistent degradation of the extracellular matrix (ECM). At the same time, atrophy of the subcutaneous fat layer reduces mechanical cushioning and support. These structural changes collectively manifest as classic aging hallmarks such as wrinkles, laxity, and delayed repair. Among these, fibroblasts play a pivotal role in cellular aging and skin functional decline due to their dominance in ECM synthesis and remodeling. Notably, despite similar phenotypes, their underlying mechanisms differ markedly: intrinsic aging is primarily regulated by temporal and genetic factors, whereas extrinsic aging is dominated by environmental exposure (particularly UV radiation). The following sections will separately outline the core characteristics and molecular foundations of these two aging processes.

### 2.2. Endogenous Aging

Intrinsic aging is largely driven by the passage of time and genetic background and is considered an inevitable progressive process that generally begins to become apparent after the mid-twenties [28]. Clinically, intrinsic aging is typically manifested by fine lines, dry skin, and sagging [29]. The physiological changes in the skin associated with aging are thought to be driven by intrinsic factors such as hormonal variations, genetic predispositions, and cellular senescence, defined as a permanent stop in the cell cycle. These changes are driven by several related aging characteristics, including genomic instability, telomere degradation, epigenetic changes, and proteostasis disruption, all believed to compromise cellular renewal and skin health [30]. From a histological perspective, older skin exhibits a flattened dermal–epidermal junction (DEJ), along with fewer anchoring structures like hemidesmosomes and thinner dermal papillae. These structural alterations weaken the mechanical attachment between the epidermis and dermis, thereby increasing skin fragility. At the tissue level, these modifications result in the appearance of wrinkles and a diminished capacity for wound healing [13]. At the microstructural level, the quantities of type I collagen (COL-I) and type III collagen (COL-III), as well as their ratio, significantly influence skin elasticity and mechanical strength. These factors undergo changes with aging [31].

Endogenous aging is a progressive process driven by both genetic factors and time-dependent damage, typically becoming apparent after age 25. Clinically, it manifests as fine lines, dry skin, and generalized laxity. Histologically, it is characterized by flattening of the dermal–epidermal junction (DEJ), disorganized collagen fiber arrangement, and a reduction in total ECM volume.

### 2.3. Exogenous Aging

Extrinsic aging, or exogenous aging, is predominantly caused by environmental factors, with significant contributions from prolonged ultraviolet (UV) exposure [28,32]. Photoaging, when superimposed on intrinsic aging, is a major contributor to the clinical picture of premature skin aging, typically manifested by coarse, deep wrinkles and irregular pigmentation (such as freckle-like macules). Cumulative acute and chronic UV exposure has been associated with increased susceptibility to skin cancer [29,33,34]. Histologically, photoaged skin often shows an initial reparative response characterized by epidermal thickening together with increased melanin production. Subsequently, the dermis is characterized by deposition of abnormal elastic fibers (i.e., solar elastosis), collagen degeneration, and a distorted, dilated microvascular network [35]. The skin experiences oxidative stress at the molecular level due to external factors like UV radiation and environmental pollution, which cause an overproduction of reactive oxygen species (ROS). The accumulation of ROS is accompanied by a series of downstream events, including ECM degradation, lipid peroxidation, and DNA damage [28]. Additionally, experiments have demonstrated that UV exposure can trigger the overexpression of MMP-1 and MMP-9, which collectively accelerate collagen degradation in the ECM [36]. Among these enzymes, MMP-1 preferentially cleaves the triple-helix structure of type I collagen, thereby promoting fragmentation of collagen fibers. Concurrently, MMP-9 (gelatinase) contributes to the further degradation of collagen fragments [37,38,39].

Exogenous aging primarily stems from prolonged ultraviolet exposure, which induces oxidative stress and DNA damage, upregulates matrix metalloproteinases (such as MMP-1 and MMP-9), accelerates degradation of type I and III collagen, promotes abnormal elastic fiber deposition (i.e., elastic fiber degeneration), and causes microvascular structural distortion. These alterations collectively manifest as deep wrinkles, skin roughness, uneven pigmentation, and increased skin cancer risk. When extrinsic factors compound endogenous aging processes, the skin’s phenotypic aging significantly accelerates, underscoring the critical role of daily photoprotection in delaying skin aging.

## 3. Mechanisms of Fibroblast Influence on Skin Aging

**Figure 1:**
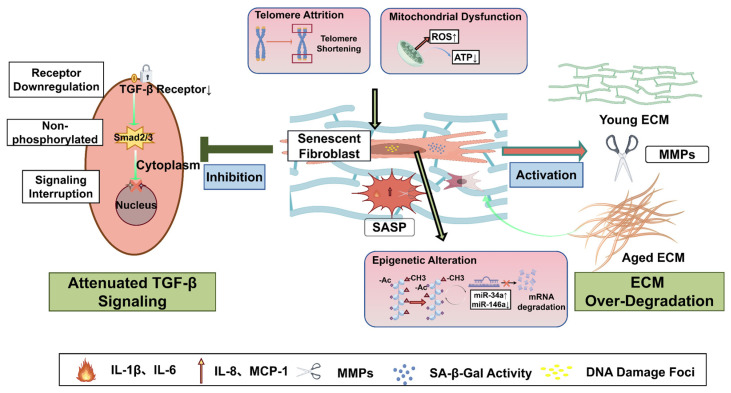
Mechanisms of fibroblast senescence and extracellular matrix dysregulation in skin aging. In the aged dermis, intrinsic stressors such as telomere attrition and mitochondrial dysfunction (leading to elevated ROS and mtDNA damage) trigger dermal fibroblasts to enter a stable senescent state. This state is characterized by persistent DNA damage foci, increased SA-β-gal activity, and the constitutive secretion of the pro-inflammatory SASP. The SASP comprises cytokines (e.g., IL-1β, IL-6), chemokines (e.g., MCP-1, IL-8), and MMPs. Concurrently, senescent fibroblasts exhibit attenuated TGF-β signaling due to receptor downregulation and impaired Smad2/3 phosphorylation. This SASP, reinforced by associated epigenetic alterations (e.g., histone methylation, dysregulation of miRNAs such as miR-34a), drives chronic inflammation, immune cell recruitment, and excessive degradation of the ECM. The resultant shift from a structured, youthful ECM to a fragmented and disorganized aged ECM leads to loss of skin integrity, atrophy, and the classic signs of skin aging. In this figure, upward arrows indicate upregulation or increased levels, and downward arrows indicate downregulation or decreased levels of the indicated molecules. This figure is created by figdraw.

Fibroblasts play a crucial role in maintaining dermal matrix homeostasis, and their functional decline is closely associated with skin aging. Recent studies reveal that fibroblasts are not a homogeneous population; papillary and reticular layer subpopulations exhibit distinct response patterns during aging. Multiple stressors (such as oxidative damage and telomere shortening) can induce stable cell cycle arrest through pathways like the DNA damage response. During this process, mitochondrial dysfunction frequently emerges as a concomitant feature or feedback amplifier, further exacerbating oxidative stress. Concurrently, epigenetic mechanisms—including alterations in DNA methylation, histone modification rearrangements, and non-coding RNA regulation—undergo extensive remodeling, facilitating the conversion of initial stress signals into long-term, stable aging-associated phenotypes. Aging fibroblasts also disrupt the synthetic-degradative homeostasis of the extracellular matrix by secreting SASP, disrupting MMP/TIMP balance, and attenuating TGF-β signaling, thereby exacerbating local microenvironmental disruption (Figure 1).

### 3.1. Core Drivers and Molecular Hubs of Fibroblast Senescence

Fibroblasts, the most abundant cells in the dermis, are the primary producers of collagen and elastic fibers [40,41]. Consequently, the functional state of fibroblasts is considered a major determinant of the skin aging phenotype. Comparative analysis has demonstrated that skin from individuals older than 80 years has about 35% fewer fibroblasts compared to those aged 18–29, but a higher proportion of these cells are senescent [42,43]. Compared to young fibroblasts, aged fibroblasts exhibit significant phenotypic alterations, such as a 28–79% increase in cell surface area, pseudopod perimeter, number, and total length, along with nuclear surface area, and a decrease in cell roundness [44]. Fibroblast senescence is driven by a variety of factors and the interaction of different molecular pathways. Irreversible replicative senescence and stress-induced senescence, where reactive oxygen species (ROS) are key, are typically seen as the primary drivers. Mitochondrial dysfunction has been proposed to function as an important molecular hub, linking these distinct drivers and amplifying senescence-related signaling.

#### 3.1.1. The Heterogeneity of Fibroblasts Determines Their Differential Fates During the Aging Process

Traditional research often regarded dermal fibroblasts as functionally homogeneous units of matrix synthesis. However, recent single-cell RNA sequencing studies have clearly revealed their high heterogeneity. At least two major functional subpopulations exist in human dermis: papillary fibroblasts (Fp) located in the superficial dermis and reticular fibroblasts (Fr) situated in the deep dermis [45,46]. The two are not only separated anatomically, but also have fundamental differences in developmental origins (such as HOX gene expression profiles), proliferative potential, metabolic characteristics, and baseline activity of signaling pathways [45,47]. This intrinsic identity program profoundly influences their sensitivity and response patterns to the core drivers of aging, including oxidative stress, replicative stress, and mitochondrial dysfunction. Therefore, the papillary layer subpopulation primarily contributes to aging through disruption of the vascular microenvironment, while the reticular layer subpopulation drives the decline in skin structure and function mainly via ECM imbalance and fibrosis.

#### 3.1.2. Oxidative Stress and Replicative Aging: The Two Core Drivers of Aging

Oxidative stress is widely regarded as a common molecular basis for both environmental aging (such as photoaging) and intrinsic aging. As byproducts of cellular metabolism, reactive oxygen species (ROS) are generated and maintained in a dynamic equilibrium under physiological conditions [48]. With advancing age or in the presence of external stressors such as ultraviolet (UV) radiation, organelles such as mitochondria become major sources of excessive ROS, leading to the accumulation of oxidative damage [49]. Excessive ROS have been reported not only to cause DNA damage directly (including damage to nuclear and mitochondrial DNA) but also to activate the NF-κB signaling pathway, upregulate matrix metalloproteinase-9 (MMP-9), inhibit collagen synthesis, and induce the senescence-associated secretory phenotype (SASP), ultimately contributing to cellular functional decline [50,51].

Replicative senescence is believed to function as a natural ‘division clock’ within cells, as telomeres, which protect chromosome ends, gradually shorten with each cell division [52]. When telomeres become critically short, the p53–p21–RB pathway activates a persistent DNA damage response, leading cells to an irreversible halt in proliferation [53,54]. Importantly, oxidative stress and replicative senescence do not occur in isolation. The telomeric region, which is guanine-rich (G-rich) and has a relatively low redox potential, is particularly susceptible to attack by ROS. This susceptibility results in oxidative lesions such as 8-oxoG formation, which accelerate telomere shortening and disrupt the function of the shelterin complex. Through the ATM/ATR–p53 pathway, these processes are thought to act together to accelerate the aging process [55,56]. In summary, oxidative stress not only directly damages biomolecules but also accelerates telomere attrition, thereby creating a synergistic amplification effect between replicative aging and stress-induced aging.

#### 3.1.3. Mitochondrial Dysfunction: An Amplifier of Aging and Trigger of Inflammation

Mitochondrial dysfunction is increasingly regarded as an important hub that connects multiple drivers of cellular aging. In aged fibroblasts, mitochondrial morphological abnormalities (such as increased organelle size and disorganized cristae architecture) are commonly observed, together with elevated mitochondrial reactive oxygen species (mtROS), decreased ATP production, reduced mitochondrial membrane potential, diminished mitochondrial DNA (mtDNA) copy number, and, in some settings, reduced intramitochondrial mtROS levels [57]. Dysfunctional mitochondria are therefore thought to contribute to aging-related phenotypes through multiple mechanisms.

Reactive oxygen species (ROS) originating from mitochondria can cause oxidative harm to mtDNA, resulting in oxidized mtDNA (ox-mtDNA) escaping from the mitochondrial matrix into the cytoplasm [58]. The cGAS–STING signaling axis is activated when cyclic GMP–AMP synthase (cGAS) detects cytoplasmic ox-mtDNA [59,60]. STING activation leads to the recruitment and activation of TBK1 and IRF3, which in turn induces the production of SASP factors, including type I interferons, playing a role in establishing chronic inflammation [61]. In addition, STING activation has been shown to further increase ROS production (for example, through disruption of iron metabolism or mitochondrial damage), forming a ROS–cGAS–STING–ROS positive feedback loop that can exacerbate aging. Mitochondrial dysfunction (such as membrane potential depolarization) and ROS themselves have also been identified as potent activators of the NLRP3 inflammasome [62]. Furthermore, ox-mtDNA fragments (such as those generated by the FEN1 endonuclease) can directly bind NLRP3, triggering inflammasome assembly and interleukin-1β (IL-1β) release [63].The activation of the NLRP3 inflammasome results in the maturation and release of proinflammatory cytokines like IL-1β, which in turn heightens local inflammatory responses [64,65]. As we age, a decrease in intracellular NAD^+^ levels has been noted to hinder the function of deacetylases like SIRT3. Sirtuins, including SIRT1 and SIRT3, play crucial roles in managing oxidative stress responses, mitochondrial biogenesis, and genomic stability [66,67]. Reduced sirtuin activity is associated with a broad reduction in cellular stress resistance, thereby contributing to a self-perpetuating cycle of “mitochondrial dysfunction–oxidative stress–inflammation–energy crisis.” This cycle is thought to drive fibroblast aging and to accelerate its progression [68,69,70].

In summary, core drivers such as oxidative stress, replicative senescence, and mitochondrial dysfunction are believed to initiate the senescence program in fibroblasts by directly damaging biomolecules and activating specific signaling pathways. However, these transient signals are ultimately translated into stable and persistent changes in gene expression that lock cells into an irreversible senescent state. This transition is thought to depend largely on extensive reprogramming of epigenetic mechanisms. Consequently, mitochondrial dysfunction establishes a self-reinforcing “oxidative stress–inflammation–energy depletion” cycle through the mtROS–cGAS–STING–NLRP3 axis and reduced sirtuin activity, continuously driving fibroblast senescence.

### 3.2. Epigenetic Regulatory Network of Fibroblast Senescence

**Figure 2:**
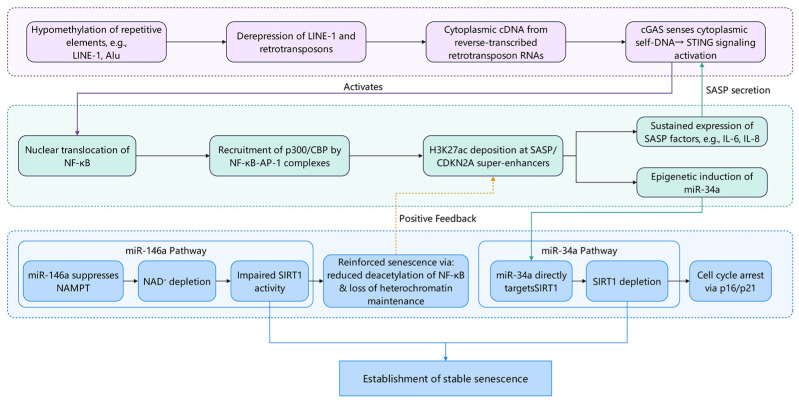
A hierarchical epigenetic cascade locks fibroblasts into a self-sustaining senescent state. Global DNA hypomethylation during aging derepresses retrotransposons—chiefly LINE-1, with Alu elements activated in trans—yielding cytoplasmic cDNA via reverse transcription. This endogenous DNA engages cGAS, activating STING and downstream NF-κB/AP-1. These factors recruit p300/CBP to acetylate H3K27 at super-enhancers of SASP loci (IL6, IL8), sustaining their transcription. Although CDKN2A/p16 lacks canonical super-enhancer control, its expression is amplified by the SASP-driven inflammatory niche and Polycomb complex eviction. Parallel NF-κB–dependent induction of miR-34a and miR-146a enforces senescence: miR-34a silences SIRT1 and CDK4/6, while miR-146a represses NAMPT, depleting NAD^+^ and further inactivating SIRT1. Collectively, DNA methylation loss, enhancer hyperacetylation, and miRNA-mediated feedback converge to encode a durable epigenetic memory of senescence.

Fibroblasts undergo extensive epigenetic alterations during aging, including reprogramming of DNA methylation patterns, abnormal histone modifications, and dysregulation of non-coding RNA expression. Epigenetic alterations during fibroblast senescence are not isolated events but constitute a hierarchical cascade network initiated by DNA methylation disruption, mediated by histone modifications that transform chromatin states, and regulated by non-coding RNAs that lock functional outputs. Research indicates these epigenetic layers interact through inflammatory signaling and transcription factor feedback loops, collectively constructing a self-sustaining memory of senescence (Figure 2).

#### 3.2.1. DNA Methylation: Genomic Stability and Dysregulation of Innate Immunity

Aging fibroblasts have been reported to exhibit a pattern of genome-wide hypomethylation coexisting with hypermethylation in specific gene promoter regions. Retrotransposons (e.g., LINE-1 and Alu) can be activated by promoter hypomethylation. Their transcripts can be reverse-transcribed into cDNA, accumulate in the cytoplasm, and be sensed by cyclic GMP–AMP synthase (cGAS), thereby reactivating the cGAS–STING pathway. This mechanism functionally links epigenetic dysregulation to innate immune activation [71]. Patterns of DNA methylation have been widely used as the basis for so-called “epigenetic clocks”. During aging, the genome shows a broad trend toward hypomethylation [72,73]. This hypomethylation can aberrantly reactivate transposons that were previously silenced, such as LINE-1 and Alu elements [74]. One important downstream consequence is that the ORF2p protein encoded by activated LINE-1 has reverse transcriptase activity, enabling it to reverse-transcribe both its own RNA transcripts and those of Alu elements into cDNA [75]. This process can result in abnormal accumulation of such cDNAs in the cytoplasm. These “self” cDNAs are recognized by the cytoplasmic DNA sensor cGAS, in a manner analogous to viral DNA, thereby inappropriately activating the cGAS–STING signaling pathway. This process activates a type I interferon response and chronic inflammation that is associated with aging, termed ‘inflammaging’ [71]. According to studies conducted by Glück et al., the rupture of the nuclear envelope during aging can cause chromatin fragments to move into the cytoplasm, where they are identified by cGAS and trigger the STING pathway. This, in turn, induces a type I interferon response and the production of an inflammatory SASP, ultimately supporting the maintenance of both cell-autonomous and non-cell-autonomous aspects of cellular aging and contributing to tissue functional decline [76]. Therefore, targeting the cGAS–STING signaling pathway activated downstream of LINE-1 and Alu elements has been proposed as a potential strategy for developing anti-aging interventions. Additionally, LINE-1 hypomethylation-induced activation of the cGAS–STING pathway and downstream type I interferon and NF-κB signaling has been reported to promote the recruitment of acetyltransferases such as p300 to specific loci. This alters H3K27ac modification levels and upregulates the expression of aging-related non-coding RNAs, including miR-34a [77,78]. Thus, genome-wide hypomethylation serves not only as a marker of genomic instability but also as a key upstream event that initiates downstream histone reprogramming.

#### 3.2.2. Histone Modifications and Chromatin Remodeling: The Switch for Gene Expression Programs

Under the synergistic action of NF-κB and AP-1, significant H3K27ac enrichment occurs in the enhancer regions of aged fibroblasts, particularly in regulatory zones encoding SASP factors such as IL-6 and IL-8, as well as cell cycle suppressor genes like CDKN2A/p16. These histone modification alterations are closely associated with the activation and inactivation regulation of aging-related genes. Studies indicate that a prominent epigenetic feature of senescent fibroblasts is the enrichment of histone H3 lysine 27 acetylation (H3K27ac) in enhancer and super-enhancer regions of inflammation- and aging-related genes (such as SASP factors and CDKN1A), thereby driving their sustained high expression. This change primarily arises from the AP-1 transcription factor recruiting histone acetyltransferase p300 to catalyze localized H3K27 acetylation. Under physiological conditions, this process is inhibited by HDAC4, which undergoes proteolytic degradation during aging, thereby releasing the repressive control over these enhancers [78]. Meanwhile, reduced levels of Class IIa histone deacetylases (such as HDAC4) may indirectly amplify the activity of the AP-1 pathway [78,79]. In young cells, FOXM1 functions as an important transcription factor that supports proliferation, and H3K27ac at its enhancer region is associated with its high expression. Studies have suggested that H3K27ac can enhance transcriptional activity at the FOXM1 promoter through an HMGCL-mediated mechanism, thereby helping to sustain its expression levels [80]. The reduction of this modification with age has been connected to lower FOXM1 levels, which eases the inhibition of AP-1 transcription factors such as c-JUN, thereby enabling the expression of many genes associated with aging and inflammation [81]. Notably, the AP-1/p300 complex can also bind to the miR-34a promoter region, directly driving its transcription and extending chromatin-level reprogramming to post-transcriptional regulation.

#### 3.2.3. Non-Coding RNAs: The Fine-Tuned Layer of Post-Transcriptional Regulation

The expression of non-coding RNAs is itself regulated by upstream epigenetic events. For example, the miR-34a locus exhibits H3K27me3 suppression and promoter hypermethylation in young cells. During aging, NF-κB/p300-mediated H3K27ac enrichment and localized demethylation jointly lift this suppression, leading to its significant upregulation [77,78,82].

Non-coding RNA (ncRNA) has garnered increasing attention as a key regulator of fibroblast homeostasis. Studies indicate that miR-146a, whose expression rises during aging, directly targets the 3′-UTR of the NAMPT gene. This inhibition suppresses NAMPT protein expression, leading to decreased NAD^+^ levels and reduced SIRT1 activity, thereby impairing the function of anti-aging pathways such as AMPK [83]. Similarly, miR-34a is significantly upregulated in fibroblasts under oxidative stress or DNA damage conditions. By inhibiting targets such as SIRT1 and CDK4/6, it promotes cell cycle arrest and the establishment of senescence phenotypes [82]. In summary, epigenetic changes during fibroblast senescence may follow a hierarchical sequence: genome-wide hypomethylation (e.g., de-repression of LINE-1 elements) leads to cytoplasmic DNA accumulation, which in turn activates the cGAS–STING pathway and downstream NF-κB signaling; The latter, by recruiting acetyltransferases such as p300, promotes H3K27ac modification and transcriptional activation at specific loci (including SASP factors and non-coding RNAs like miR-34a); these non-coding RNAs further suppress key regulatory factors like SIRT1 or NAMPT, impairing cellular adaptation to oxidative or metabolic stress, thereby contributing to the maintenance of the senescence phenotype.

### 3.3. Molecular Mechanisms of Imbalance in ECM Synthesis and Degradation

The youthful state of the dermis is maintained by a balance between ECM synthesis and degradation. With age, this balance becomes progressively imbalanced, featuring diminished synthesis and elevated degradation of structural proteins such as collagen. This imbalance is thought to arise from the combined effects of cell-autonomous changes, alterations in the secretome, dysregulated signaling pathways, and deterioration of the physical microenvironment.

#### 3.3.1. Senescent Cell Accumulation and SASP: The Engine of ECM Dysregulation

Accumulation of senescent fibroblasts in both in vivo and in vitro settings is thought to play a crucial role in ECM imbalances. An increase in these fibroblasts has been noted with aging. These accumulated cells secrete factors collectively referred to as the SASP, which include multiple interleukins, chemokines, proteases, and growth factors [9,84,85]. Through paracrine mechanisms, SASP components are thought to induce neighboring cells to enter a senescent state, thereby generating persistent inflammatory signaling and amplification within the tissue microenvironment [9,86]. During skin photoaging, for example, ultraviolet B (UVB) radiation has been shown to upregulate and activate PAR2. Through the Gαq–PLC–PI3K/Akt signaling axis, PAR2 activation promotes phosphorylation and activation of NF-κB, enhancing the expression of SASP factors such as IL-6 and IL-1β and thereby contributing to chronic inflammation, while at the same time promoting phosphorylation and inactivation of FoxO6 (Ser184), which reduces its regulation of antioxidant genes such as MnSOD. This sequence of events favors ROS accumulation and exacerbates oxidative stress [87]. It is thought that the advancement of cutaneous photoaging is driven by the combination of oxidative stress and inflammatory activation. EPDR1 has been recently recognized as a new element of the SASP in recent research that can mediate the toxicity of senescent cells toward young fibroblasts, thereby reducing their capacity for ECM synthesis. Furthermore, disruption of ECM structure itself can promote fibroblast senescence, as collagen fragments impair normal cell–matrix interactions. For instance, studies have shown that genetic deletion of collagen-binding integrins (α1β1, α2β1, and α11β1) markedly impairs dermal fibroblast function [88]. According to research by Selman and Pardo, cumulative damage to the ECM during aging—such as collagen cross-linking and glycation—has been proposed to lead to matrix stiffening. This, in turn, is thought to activate fibroblasts and promote their differentiation into myofibroblasts through mechanotransduction pathways (e.g., integrin–YAP/TAZ), while also inducing cellular senescence and a SASP phenotype. The resulting SASP releases factors such as TGF-β and matrix metalloproteinases (MMPs), which further disrupt ECM structure and mechanical balance, establishing a self-reinforcing “fibrosis–senescence” cycle [89]. Moreover, the ECM is considered not only a consequence of cellular senescence but also an active participant in driving aging. In the context of vascular system research, Sox9 has been identified as a transcription factor involved in a positive feedback loop that facilitates the aging of vascular smooth muscle cells by adjusting their structural and mechanical properties [90]. Therefore, SASP not only directly degrades the ECM but also disrupts cell–matrix mechanical signaling feedback, forming a vicious cycle where ECM damage induces fibroblast senescence, which in turn leads to increased SASP production.

#### 3.3.2. Dysregulation of the Proteasome Network: Overactivation of the Degradation System

The regulation of ECM degradation is largely believed to depend on the balance between MMPs and their tissue inhibitors (TIMPs). Aged fibroblasts have been reported to express and secrete increased amounts of MMP-1, MMP-3, and related enzymes, which can directly degrade the ECM [9,91]. Chronic inflammatory states see SASP factors like IL-8 and IL-1β enhancing MMP expression via the NF-κB pathway, which promotes ECM degradation. Similarly, experimental findings have revealed that inhibiting NF-κB is connected to reduced amounts of MMP-1, MMP-8, and MMP-9. In patients suffering from bronchiectasis, Rothia mucilaginosa has been found to diminish inflammatory reactions by blocking the NF-κB pathway, which results in reduced expression of IL-8, IL-1β, and several MMPs [92]. These findings support an important role for NF-κB in the regulation of inflammation-induced tissue remodeling. In addition, TIMP-1, an endogenous inhibitor of MMPs such as MMP-9, is thought to limit ECM degradation. As a result, reducing TIMP-1 expression could speed up ECM breakdown and contribute to the emergence of age-related diseases [93]. Consequently, downregulation of TIMP-1 expression is expected to increase the MMP/TIMP ratio, thereby facilitating ECM degradation. Therefore, NF-κB-mediated MMP/TIMP imbalance represents the core mechanism underlying uncontrolled ECM degradation in the inflammatory microenvironment.

#### 3.3.3. TGF-β Signal Attenuation: Insufficient Power in the Synthesis Engine

Collagen and elastin synthesis in fibroblasts is significantly regulated by the TGF-β/Smad signaling pathway. In young fibroblasts that function normally, TGF-β has been demonstrated to trigger the expression of Engrailed 1 (EN1) via a pathway dependent on SMAD3. EN1 has later been identified as a molecular amplifier of TGF-β signaling, playing a role in cytoskeletal reorganization and facilitating myofibroblast differentiation and excessive extracellular matrix buildup, thus contributing to skin in fibrosis [94,95]. In summary, decreased activity of the TGF-β/Smad pathway leads to impaired collagen synthesis, preventing ECM remodeling from compensating for ongoing degradation and ultimately resulting in dermal structural collapse.

## 4. Senescent Fibroblasts Drive Multilayer Skin Degeneration Through Intercellular Signaling

**Figure 3:**
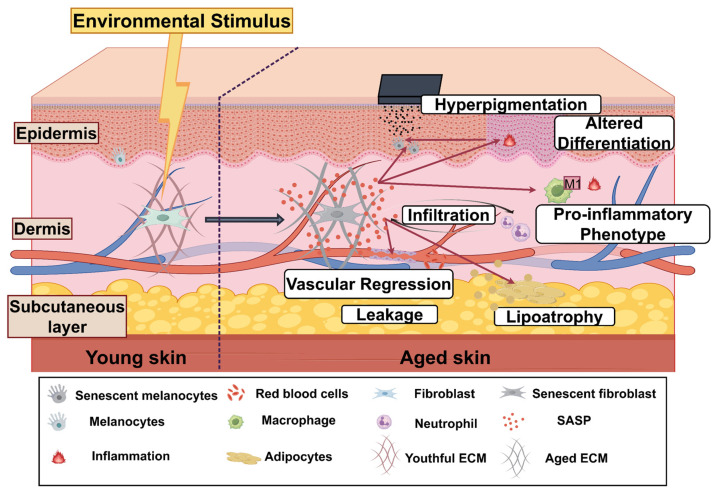
The central role of cellular senescence and the SASP in driving skin aging and dysfunction. The structural and functional decline of aging skin is driven by the accumulation of senescent dermal fibroblasts. These cells secrete a potent pro-inflammatory mixture termed the SASP, which orchestrates multi-tissue damage. Key SASP-mediated pathological processes include: (1) induction of chronic inflammation, immune cell infiltration, and polarization of macrophages toward a tissue-damaging M1 phenotype; (2) fragmentation and stiffening of the ECM, leading to loss of skin integrity and elasticity; (3) regression of cutaneous vasculature and increased endothelial leakage, compromising nutrient delivery and metabolic waste clearance; and (4) disruption of epidermal homeostasis through altered keratinocyte differentiation and hyperactivity of melanocytes, resulting in clinical hyperpigmentation. Collectively, these cascading events lead to the hallmark phenotypes of aged skin, including dermal and subcutaneous atrophy (lipoatrophy) and impaired regenerative capacity. This figure is created by figdraw.

Aging dermal fibroblasts not only exhibit diminished synthetic and reparative capacities themselves but also exert widespread effects on neighboring cells through paracrine factors, extracellular vesicles, and matrix remodeling. They can compromise the barrier function of keratinocytes, disrupt the distribution and activity of melanocytes, and promote the pro-inflammatory polarization of macrophages, thereby exacerbating localized chronic inflammation. Furthermore, abnormal matrix components and proteases secreted by senescent fibroblasts can compromise microvascular integrity, indirectly affecting subcutaneous tissue homeostasis. These cross-cell interactions collectively drive the synergistic degradation of the skin’s multilayered structure (Figure 3). However, the aforementioned cross-cell effects do not originate from a functionally homogeneous population of fibroblasts. Fp and Fr exhibit distinctly different secretory profiles and stress responses during aging. Fp primarily secrete VEGF, IGF-1, and Angiopoietin-1 to maintain superficial microvascular homeostasis. However, with advancing age, their SASP progressively shifts toward pro-inflammatory factors, leading to vascular dysregulation [96]. Conversely, Fr primarily synthesize HGF, FGF-21, and type I collagen. Their aging is often accompanied by NRF2 pathway inhibition, resulting in reduced antioxidant capacity, decreased ECM synthesis, and enhanced release of pro-fibrotic SASP factors like TGF-β [96,97]. Thus, Fp functional decline primarily indirectly impacts epidermal and immune homeostasis through microvascular disruption, while Fr abnormalities directly drive deep ECM degradation and tissue stiffening. The synergistic degradation of skin’s multilayered structure fundamentally results from the combined effects of these two subpopulations through distinct mechanisms.

### 4.1. Disruption of Epidermal Homeostasis

Aging dermal fibroblasts regulate the function of keratinocytes and melanocytes by remodeling the microenvironment and releasing paracrine signals, leading to skin barrier impairment and abnormal pigmentation. Among these, Fp located in the superficial layer adjacent to the epidermal basement membrane play a pivotal role in maintaining epidermal homeostasis due to their advantageous anatomical position [98]. This regulation is primarily achieved through two complementary mechanisms: soluble SASP factors (such as IL-6 and MMPs) and senescence-associated extracellular vesicles (SA-EVs). The latter are now widely recognized as the insoluble component of SASP, capable of transmitting senescence signals between cells by selectively loading pro-senescence molecules [99]. The targeting of EVs to epidermal cells is partially determined by their surface protein characteristics—for example, vesicles carrying integrin α6β4 are more readily internalized by keratinocytes and melanocytes [100]. Aging dermal fibroblasts secrete large quantities of vesicles enriched with regulatory miRNAs such as miR-10a, miR-30c, and miR-451a. Upon entering recipient cells, these miRNAs suppress target genes like EPHA4 and RUNX2, activate the p53/p21 pathway, exacerbate mitochondrial dysfunction and oxidative stress, ultimately inducing paracrine-mediated senescence in keratinocytes and melanocytes [101]. Therefore, EVs not only expand the functional scope of SASP but also enable precise regulation of the epidermis through molecular recognition.

#### 4.1.1. Keratinocyte Barrier Dysfunction

Keratinocyte homeostasis is thought to depend strongly on the underlying dermal microenvironment. Senescent fibroblasts are believed to disrupt this homeostasis through both structural and paracrine mechanisms. As skin ages, the DEJ becomes less pronounced, and the structural strength of anchoring systems, including hemidesmosomes, diminishes, causing reduced adhesion between the dermis and epidermis. This alteration, together with reduced resistance of the collagen fiber network (e.g., an increased type III/I collagen ratio and fewer elastic fibers), is thought to diminish the skin’s mechanical resistance to shear forces and pressure [13]. An indirect mechanism is thought to arise from breakdown of the extracellular matrix. Research indicates that senescent fibroblasts gain a pro-fibrotic characteristic, enabling them to alter the ECM. These cells can degrade collagen and elastic fibers by secreting molecules such as MMPs, while simultaneously promoting aberrant ECM deposition—a mechanism that has been documented in multiple fibrosis and cancer models [102]. Aberrant ECM deposition can disrupt the architecture of the DEJ and compromise its structural integrity. This not only weakens the skin’s mechanical anchoring function but also interferes with cellular sensing of mechanical and biochemical cues transmitted by the ECM. Consequently, the normal differentiation program of keratinocytes is impaired and skin barrier function is compromised [103]. In addition, more direct paracrine effects have been described. Aged fibroblasts can send signals that promote aging and fibrosis through their SASP, changing the tissue environment, diminishing the regenerative ability of epidermal cells, and encouraging further aging. The SASP is regarded as an important mediator of non-cell-autonomous paracrine signaling by senescent cells, inducing neighboring cells to adopt a senescent state and thereby amplifying senescence within the tissue [86]. In vivo experiments have provided supporting evidence for these interactions. According to research by Franco et al., transplantation of aged fibroblasts into the dermis of young mice has been shown to result in an increased number of p16INK4A-positive keratinocytes in the epidermis above the graft site, and increased infiltration of CD45^+^ leukocytes and CD68^+^ macrophages has been observed in the dermis [85]. These findings suggest that aged fibroblasts can establish a chronic inflammatory microenvironment in vivo that cooperatively compromises skin integrity. Thus, senescent fibroblasts disrupt keratinocyte homeostasis through dual mechanisms—loss of mechanical anchoring and SASP-mediated paracrine signaling—thereby initiating epidermal-dermal synergistic aging.

#### 4.1.2. Melanocyte Hyperactivation and Pigmentary Dysregulation

Age-related pigmented spots (such as senile lentigines) have been suggested to arise primarily not from intrinsic aging of melanocytes themselves but from pathological remodeling of signaling derived from senescent dermal fibroblasts. Recent studies have identified several paracrine signaling axes that may contribute to this process. Research indicates that under photoaging conditions, the presence of advanced glycation end products (AGEs) in aging fibroblasts leads to an increase in the RNA-binding protein YTHDF2. YTHDF2 promotes degradation of mRNA encoding the deubiquitinating enzyme A20, thereby reducing A20 protein levels. The absence of A20 removes the suppression of the NF-κB/NLRP3 inflammasome pathway, leading to a rise in cytokine production like IL-18. Ultimately, this paracrine activation is thought to stimulate melanocytes to increase melanin production [104]. Another pathway of interest involves Hippo–YAP signaling. It has been found in studies that YAP/TAZ co-activators are less active in fibroblasts that have aged intrinsically [105]. In other settings, YAP activation has been shown to upregulate DKK1 expression through Src kinase, thereby inhibiting the Wnt/β-catenin signaling pathway [106]. In aged fibroblasts, reduced YAP activity decreases DKK1 secretion, thereby relieving this inhibition and promoting β-catenin nuclear translocation. This in turn upregulates expression of microphthalmia-associated transcription factor (MITF) and enhances tyrosinase activity, thereby stimulating synthesis of the melanin precursor L-DOPA. Ultimately, these changes are thought to drive melanocyte proliferation and pigment synthesis [107,108,109]. In summary, senescent fibroblasts may drive melanocyte activation, directly or indirectly (via keratinocytes), through multiple parallel paracrine pathways, including the YTHDF2/A20/IL-18 axis and the YAP/DKK1/Wnt axis. Consequently, targeting the fibroblast secretome, rather than melanocytes themselves, has been proposed as a potential therapeutic strategy for age-related hyperpigmentation. In summary, senescent fibroblasts indirectly activate melanogenesis pathways through paracrine axes such as YTHDF2/A20/IL-18 and YAP/DKK1/Wnt, revealing the dermal origin of abnormal pigmentation.

### 4.2. Reprogramming of the Immune Microenvironment

Aging fibroblasts are thought to shape the local microenvironment into a chronic, low-grade inflammatory state—often referred to as “inflammaging”—through complex bidirectional interactions with innate immune cells, particularly macrophages and neutrophils. This state is considered an important mechanism that contributes to degeneration of skin tissue.

#### 4.2.1. Macrophage Polarization and Immune Evasion

Macrophages are widely regarded as important mediators that amplify inflammation within the aging microenvironment. Senescent fibroblasts have been reported to secrete chemokines such as CCL2 and CCL5, which recruit circulating monocytes [110]. In the presence of SASP factors (such as GM-CSF), these macrophages are polarized toward a pro-inflammatory M1 phenotype and secrete increased levels of IL-1β, TNF-α, and IL-6 [111,112]. These inflammatory mediators are thought to both induce senescence in neighboring fibroblasts and suppress their collagen synthesis, thereby contributing to disruption of dermal structure. Thus, the SASP is thought to interact with macrophage-derived inflammatory mediators, forming a “senescence–recruitment–polarization–inflammation” positive feedback loop that contributes to progressive deterioration of the microenvironment. Aging itself has been associated with altered function of tissue-resident macrophages. Macrophages in aged individuals tend to display a more pro-inflammatory profile and an imbalanced M1/M2 ratio, with increased pro-inflammatory M1 and reduced anti-inflammatory M2 populations, changes that may intensify inflammatory responses and contribute to tissue aging [113]. Senescent fibroblasts may evade immune clearance through active mechanisms. With advancing age, macrophage-mediated clearance of senescent cells (immune surveillance) is thought to decline. Concurrently, it has been observed that senescent fibroblasts exhibit higher amounts of the “don’t eat me” signal CD47 on their surface. CD47 binds to SIRPα on macrophages, sending inhibitory signals that restrict phagocytosis [114]. These observations have been proposed to provide a rationale for combined therapeutic approaches in which mTOR inhibitors (such as rapamycin) are used to attenuate SASP-associated inflammation, while CD47-blocking antibodies are used to enhance the phagocytic function of macrophages, potentially improving clearance of senescent cells. Although reticular layer fibroblasts (Fr) exhibit robust survival capacity and maintain stable density under chronic UV exposure, their microenvironment is characterized by significant immune cell infiltration, suggesting alterations in the deep dermal inflammatory environment [115].

#### 4.2.2. Neutrophil-Mediated Tissue Damage via NETosis

Neutrophils have traditionally been associated with acute inflammation, but accumulating evidence suggests that they also contribute to chronic processes relevant to skin aging. Favaretto et al. reported that aged fibroblasts can act as neutrophil chemotactic sources by secreting SASP factors such as IL-8 and serum amyloid A1, thereby continuously recruiting and activating neutrophils in the dermis [116]. In response to inflammatory stimuli, neutrophils have been demonstrated to release neutrophil extracellular traps (NETs) as they age. These NETs primarily consist of decondensed chromatin together with granular proteins such as myeloperoxidase (MPO) and neutrophil elastase (NE). These proteases may break down elements of the extracellular matrix (ECM), and neutrophil elastase in particular can effectively hydrolyze collagen and elastin, thereby jeopardizing the structural integrity of the dermis. Studies of chronic wounds, such as diabetic foot ulcers, have shown that excessive NET accumulation can exacerbate local inflammatory responses and impair tissue repair by suppressing fibroblast function and disrupting tissue-remodeling processes [117]. Therefore, aberrant activation of NETs is thought to contribute to disruption of skin tissue architecture and to promote tissue degeneration under specific pathological conditions. Consequently, neutrophils may not only mediate effects downstream of SASP signaling but also serve as independent sources of inflammation. Consequently, senescent fibroblasts continuously recruit neutrophils through SASP factors such as IL-8. The released NETs further degrade the ECM and impede repair, transforming acute effects into chronic tissue damage.

### 4.3. Collapse of Structural Support Systems

#### 4.3.1. Microvascular Degeneration

The breakdown of the dermal microvascular network is suggested as an early indicator of skin aging and seems to be closely associated with fibroblast aging, creating a harmful cycle. Fibroblasts and other senescent cells secrete a SASP containing numerous pro-inflammatory cytokines. The SASP is believed to not only attract immune cells and initiate chronic inflammation but also to cause nearby healthy cells, like endothelial cells, to become senescent through paracrine effects, thus increasing tissue damage [118,119]. On one hand, studies have suggested that aged fibroblasts promote basement-membrane degradation by secreting matrix metalloproteinases (such as MMP-9) in fibrotic and tumor microenvironments. At the same time, these fibroblasts can exacerbate pathological ECM remodeling through overexpression of pro-fibrotic genes such as CTHRC1. These processes collectively impair tissue structural integrity and may compromise vascular stability, leading to vascular leakage and functional abnormalities [102]. On the flip side, the ECM’s physical property changes are seen as key mediators of microvascular dysfunction. The diminished collagen production by older fibroblasts is associated with variations in ECM stiffness. This mechanical signal can be transmitted to endothelial cells via integrins such as α9β1, thereby inhibiting their proliferation and tubulogenic capacity. More intricately, aged endothelial cells have been reported to secrete lower levels of the growth factor midkine (MDK), weakening binding to the heparan sulfate proteoglycan SDC4 on epidermal basal cells. This disruption in endothelium–epithelium communication has been associated with downregulation of retinoic acid–metabolism genes (e.g., RBP1) in epidermal stem cells and with diminished stem-cell activity. Impaired epidermal function may, in turn, reduce nutrient support to the microvasculature, further accelerating microvascular degeneration and reinforcing this vicious cycle [120]. Therefore, a vicious cycle comprising fibroblast senescence, ECM stiffening, endothelial dysfunction, decreased epidermal stem-cell activity, and reduced vascular nutrient support is thought to collectively drive skin atrophy. Aging fibroblasts drive a bidirectional vicious cycle of microvascular degeneration and epidermal stem cell exhaustion through ECM remodeling, disrupted mechanical signaling, and impaired endothelium-epithelium communication.

#### 4.3.2. Hypodermal Adipose Atrophy

The dermis and subcutaneous adipose tissue have been viewed as a functional unit, and age-associated changes in fibroblasts have been suggested to contribute to fat atrophy through multiple mechanisms. Fibroblast-derived IGF-1 is considered an important mitogen for maintaining preadipocyte homeostasis [121]. Fibroblast senescence has been associated with reduced IGF-1 secretion, whereas inflammatory factors and MMPs within the SASP can alter the ECM microenvironment of adipose progenitor niches. Collectively, these alterations are believed to hinder the ability of adipose precursor cells to proliferate and differentiate, resulting in compromised adipose tissue regeneration and decreased skin volume [121,122]. In addition, hypertrophic adipocytes secrete inflammatory mediators such as TNF-α and IL-6, which can induce fibroblast senescence by activating the NF-κB pathway [123]. In the context of obesity, this inflammatory milieu has been reported to further promote senescence of adipose-derived stem cells (ADSCs) via the p38MAPK/NF-κB axis and to enhance the senescence-associated secretory phenotype [124]. Thus, senescent fibroblasts and dysfunctional adipose tissue may become engaged in a vicious cycle of inflammation, hypoxia, and senescence, collectively accelerating the decline of the skin’s supportive structures. Reduced IGF-1 secretion and the SASP-mediated inflammatory microenvironment jointly inhibit adipocyte precursor cell differentiation, while adipocyte-derived inflammation conversely promotes fibroblast senescence, forming a bidirectional positive feedback loop.

## 5. Fibroblast-Targeted Skin Aging Intervention Pathway Based on Clearance, Silencing, and Reconstruction Strategies

Recent research has increasingly focused on precision intervention strategies targeting the regulation of fibroblast function to delay or even improve skin aging, based on the core molecular mechanisms of fibroblast senescence and its cross-cell network effects. The accumulation of senescent fibroblasts during skin aging is attributed to factors such as DNA damage, telomere shortening, and oxidative stress. These cells are thought to drive chronic inflammation and extracellular matrix degradation by secreting SASP factors such as IL-6 and TGF-β. Moreover, extracellular vesicles from senescent cells have been shown to carry senescence signals, such as miRNAs and proteins, to nearby healthy cells, thus enhancing the senescent phenotype [125]. Current intervention strategies targeting this complex process primarily unfold along three interrelated directions: reducing the pathological burden of senescent cells through selective clearance, blocking paracrine damage by suppressing their harmful secretory phenotype, and promoting extracellular matrix remodeling alongside systemic rejuvenation of the dermal microenvironment.

### 5.1. Clearance Strategy: Selective Removal of Senescent Cells by Dissolving Agents

Senolytic agents have been created to specifically trigger the death of aging cells as a method of ‘clearance.’ Research indicates that using combinations of senolytic drugs like dasatinib and quercetin can selectively trigger apoptosis in senescent cells by targeting specific anti-apoptotic pathways these cells rely on, such as Ephrin, PI3Kδ, and BCL-xL, while having comparatively limited effects on normal cells [126]. Notably, the D+Q combination has been evaluated in multiple early-phase human clinical trials. For instance, in an open-label, uncontrolled pilot study involving patients with idiopathic pulmonary fibrosis, intermittent oral administration of D+Q demonstrated potential for improving patients’ physical function [127]. On the other hand, in elderly diabetic nephropathy patients, D+Q therapy has been shown to significantly reduce the burden of senescent cells in adipose tissue and skin, while decreasing levels of multiple SASP factors in the circulation [128]. However, large-scale randomized controlled trials targeting skin aging populations remain scarce, and long-term safety concerns—such as potential side effects like bone marrow suppression and immunosuppression—require systematic evaluation. Senescent cell dissolvers like D+Q can selectively eliminate accumulated senescent fibroblasts in the skin, thereby mitigating SASP-mediated inflammation and matrix degradation. Nevertheless, clinical evidence supporting their efficacy in skin anti-aging remains limited, and their long-term safety profile requires further validation.

### 5.2. Silent Strategy: SASP Suppression and the Metabolic Trade-Off with Rapamycin

In parallel, the SASP can be attenuated through “silencing” approaches, for example by using senomorphic agents such as rapamycin, which may alleviate chronic inflammation and matrix degradation [129,130]. Research indicates that long-term use of rapamycin can extend lifespan and improve multiple health indicators [131]. Despite its positive effects on lifespan and numerous health indicators, long-term rapamycin use remains associated with a dose-dependent risk of metabolic side effects, such as impaired glucose regulation, reduced insulin sensitivity, and dyslipidemia. Studies indicate that rapamycin rapidly and potently inhibits mTORC1, while long-term administration further impacts mTORC2 signaling. Its protective effects against aging primarily stem from mTORC1 inhibition, whereas mTORC2 signaling disruption may negatively affect metabolic status, physiological function, and survival rates [132]. Therefore, despite its significant anti-aging potential, systemic application requires careful consideration of metabolic risks.

While topical administration can reduce systemic toxicity, it faces significant challenges in penetrating the skin barrier. Rapamycin has a large molecular weight (914 Da) and high lipophilicity, making it difficult for conventional formulations to effectively penetrate the stratum corneum and reach the dermal layer where fibroblasts reside [133]. Recent studies have explored the use of nanocarriers (such as liposomes and microemulsions) or chemical modifications (like rapamycin derivatives) to enhance transdermal efficiency, though these approaches remain in the preclinical stage. Similarly, active ingredients like retinol and quercetin face limitations due to poor stability and low permeability, necessitating advanced delivery systems to achieve their intended effects. “Silencing” strategies such as rapamycin can alleviate skin aging-related inflammation and matrix degradation by suppressing SASP. However, their metabolic side effects and challenges in transdermal delivery limit clinical application, necessitating the development of safe and efficient topical delivery technologies.

### 5.3. Reconstruction and Remodeling Strategy: ECM Steady-State Recovery and Microenvironment Network Regulation

In principle, once the burden of senescent cells has been reduced, the focus of intervention can shift to “reconstruction and repair,” for example by activating the TGF-β/Smad signaling pathway to promote resynthesis of collagen and elastin [134]. In this context, agents such as retinoic acid and retinol have been used to enhance ECM synthetic activity [135]. Simultaneously, antioxidants and anti-inflammatory substances might aid in reducing the overactivation of proteases like MMPs. Liu et al. demonstrated that co-encapsulation of apigenin, a compound with antioxidant activity, and doxycycline, which exhibits broad-spectrum MMP inhibition and anti-inflammatory effects, within flexible liposomes significantly reduced UVB-induced activation of MMP-1 and MMP-9. This treatment was associated with reduced collagen degradation and attenuation of UVB-induced markers of skin photoaging [136]. Such nanodelivery strategies not only enhance the stability of active ingredients but, more crucially, overcome the bottleneck of low transdermal efficiency in traditional topical formulations, representing a key direction for future localized anti-aging interventions. Conceptually, the maintenance of ECM homeostasis can thus be viewed as relying on a dual approach of enhancing anabolic processes while limiting catabolic activity. More far-reaching interventions have focused on modifying the intrinsic aging program of cells, for example by supplementing NAD^+^ precursors (such as NR or NMN) with the aim of supporting energy metabolism and DNA repair capacity [137]. Recent work has also introduced the concept of “network-based regulation,” in which the dermal microenvironment is reshaped through exogenous growth factors, stem cell–derived exosomes, and their signaling molecules to improve intercellular communication and tissue regeneration at a more systemic level [138]. The success of future anti-aging interventions will hinge on the integration of multi-target synergistic regulation of the fibroblast senescence network with efficient delivery technologies, propelling skin aging from mere delay to genuine improvement.

Building upon the clearance or silencing of senescent fibroblasts, the key pathways for achieving structural and functional skin regeneration involve “reestablishing” the balance between ECM synthesis and degradation and “remodeling” the cellular microenvironment network. Advanced delivery systems serve as the core bridge connecting laboratory discoveries to clinical translation.

## 6. Conclusions and Looking Forward

Skin aging is an inevitable physiological process that accompanies the aging process, with the senescence of dermal fibroblasts playing a central role. This process is driven by multiple mechanisms, including mitochondrial dysfunction, telomere attrition, epigenetic reprogramming, and dysregulation of key signaling pathways such as Nrf2, TGF-β, and mTOR. These intrinsic alterations not only significantly impair collagen synthesis capacity but also promote excessive secretion of matrix metalloproteinases (MMPs), leading to accelerated degradation of the extracellular matrix (ECM). Furthermore, senescent fibroblasts disrupt keratinocyte function through paracrine effects mediated by the senescence-associated secretory phenotype (SASP), induce abnormal activation of local immune cells, and accelerate microvascular degeneration. This collectively establishes a persistent chronic low-grade inflammatory microenvironment within the skin. Crucially, this localized chronic inflammation not only compromises the skin’s structural integrity and function but may also serve as a significant source or driver of systemic “inflammaging,” adversely affecting overall health in the elderly.

Therefore, targeting fibroblast senescence is considered a key strategy for improving skin health in aging. Currently, interventions such as senolytics (e.g., dasatinib combined with quercetin), mTOR inhibitors (e.g., rapamycin), and retinol have demonstrated potential in preliminary studies. However, their clinical translation still faces challenges including low transdermal efficiency, insufficient cellular targeting, and unclear long-term safety. Given that fibroblast senescence inherently involves a complex network of multi-signaling pathways and dynamic interactions among multiple cell types, intervention strategies relying solely on single-target approaches are unlikely to achieve sustained and comprehensive benefits.

Future research urgently requires a systematic elucidation of the dynamic interaction mechanisms between distinct dermal fibroblast subpopulations and immune cells, vascular endothelial cells, and subcutaneous adipose tissue, based on an in-depth analysis of their functional heterogeneity. Concurrently, the development of smart delivery technologies capable of synergistically regulating cellular homeostasis and microenvironmental remodeling is essential. Notably, dermal fibroblasts are not a homogeneous population. Single-cell transcriptomics studies have clearly subdivided them into distinct subpopulations with different molecular characteristics and functional properties, such as Fp and Fr. Theoretically, these two subpopulations may exhibit differential responses to anti-aging interventions. For instance, Fp, with their robust proliferative and ECM synthesis capabilities, may be better suited for senomorphic strategies (e.g., mTOR inhibition) to preserve their regenerative potential. Conversely, Fr, which accumulate damage more readily during aging and exhibit pro-inflammatory phenotypes, may be more sensitive to senolytic drugs. Although this hypothesis of subset-specific intervention requires experimental validation, it offers a crucial framework for developing precision anti-aging therapies for the skin.

In summary, future research must transcend the broad concept of “fibroblasts” to deeply analyze the distinct fate trajectories, interaction preferences, and intervention responses of different subpopulations during aging. Only then can anti-aging strategies evolve from merely improving cosmetic phenotypes to genuinely restoring skin barrier integrity, tissue repair capacity, and homeostasis regulation—ultimately achieving precise, efficient, and safe skin anti-aging treatments.

## Data Availability

No new data were created in this study. Data sharing is not applicable to this article.

## Abbreviations

The following abbreviations are used in this manuscript:

ECM	Mextracellular matrix
SASP	Senescence-associated secretory phenotype
SA-β-Gal	Senescence-associated β-galactosidase
DEJ	Dermal–epidermal junction
MMPs	Matrix metalloproteinases
IL-6	Interleukin-6
IL-8	Interleukin-8
IL-1β	Interleukin-1 beta
TNF-α	Tumor necrosis factor-alpha
TGF-β	Transforming Growth Factor-beta
KNG1	Kininogen-1
COL-I	Type I collagen
COL-III	Type III collagen
UV	Ultraviolet
ROS	Reactive oxygen species
Fr	reticular fibroblasts
Fp	papillary fibroblasts
MMP-9	Matrix metalloproteinase-9
MMP-1	Matrix metalloproteinase-1
mtROS	Mitochondrial reactive oxygen species
mtDNA	Mitochondrial DNA
ox-mtDNA	Oxidized mtDNA
cGAS	Cyclic GMP–AMP synthase
H3K27ac	H3 lysine 27 acetylation
STING	Stimulator of interferon genes
TBK1	TANK-binding kinase 1
IRF3	Interferon regulatory factor 3
NLRP3	NACHT, LRR and PYD domains-containing protein 3
SIRT3	Sirtuin 3
HDACs	Histone deacetylases
LINE-1	Long Interspersed Nuclear Element-1
FOXM1	Forkhead box M1
HMGCL	3-Hydroxy-3-Methylglutaryl-CoA Lyase
AP-1	activator protein 1
c-JUN	jun proto-oncogene
ncRNAs	Non-coding RNAs
3′-UTR	3′-untranslated region
NAMPT	Nicotinamide phosphoribosyltransferase
AMPK	AMP-activated protein kinase
PAR2	Protease-Activated Receptor 2
Gαq	G protein alpha-q
PLC	Phospholipase C
PI3K/Akt	Phosphatidylinositol 3-kinase/protein kinase B
NF-κB	Nuclear factor kappa-B
FoxO6	Forkhead box O6
MnSOD	Manganese superoxide dismutase
SMAD3	suppressor of mothers against decapentaplegic 3
TIMPs	Tissue inhibitors
EN1	Engrailed 1
AGEs	Advanced glycation end products
DKK1	Dickkopf-1
CCL2	C-C motif chemokine ligand 2
CCL5	C-C motif chemokine ligand 5
mTOR	mechanistic target of rapamycin
SIRPα	signal-regulatory protein α
MITF	Microphthalmia-associated transcription factor
NETs	Neutrophil extracellular traps
CTHRC1	collagen triple helix repeat containing 1
MDK	Midkine
ADSCs	Adipose-derived stem cells
MITF	Transcription factor
IGF-1	insulin-like growth factor-1
